# Time-lapse 3-D measurements of a glucose biosensor in multicellular spheroids by light sheet fluorescence microscopy in commercial 96-well plates

**DOI:** 10.1038/srep37777

**Published:** 2016-11-25

**Authors:** Vincent Maioli, George Chennell, Hugh Sparks, Tobia Lana, Sunil Kumar, David Carling, Alessandro Sardini, Chris Dunsby

**Affiliations:** 1 Photonics Group, Department of Physics, Imperial College London, United Kingdom; 2 MRC Clinical Sciences Centre (CSC), Du Cane Road, London, United Kingdom; 3 Institute of Clinical Sciences (ICS), Faculty of Medicine, Imperial College London, Du Cane Road, London, United Kingdom; 4 Centre for Pathology, Faculty of Medicine, Imperial College London, United Kingdom.

## Abstract

Light sheet fluorescence microscopy has previously been demonstrated on a commercially available inverted fluorescence microscope frame using the method of oblique plane microscopy (OPM). In this paper, OPM is adapted to allow time-lapse 3-D imaging of 3-D biological cultures in commercially available glass-bottomed 96-well plates using a stage-scanning OPM approach (ssOPM). Time-lapse 3-D imaging of multicellular spheroids expressing a glucose Förster resonance energy transfer (FRET) biosensor is demonstrated in 16 fields of view with image acquisition at 10 minute intervals. As a proof-of-principle, the ssOPM system is also used to acquire a dose response curve with the concentration of glucose in the culture medium being varied across 42 wells of a 96-well plate with the whole acquisition taking 9 min. The 3-D image data enable the FRET ratio to be measured as a function of distance from the surface of the spheroid. Overall, the results demonstrate the capability of the OPM system to measure spatio-temporal changes in FRET ratio in 3-D in multicellular spheroids over time in a multi-well plate format.

Multicellular spheroids (MCS) provide a 3-D model of *in vitro* cell culture and are increasingly being used in *in vitro* assays^1^,^2^. Compared to 2-D cell monolayer culture on a plastic or glass surfaces, MCS provide cell-cell contacts and gradients of environmental parameters such as oxygen, nutrients and pH similar to those found in tumours^1^,^2^. The oxygen gradient is caused by the rate of consumption of oxygen by cells in the MCS being greater than the rate of diffusion of O_2_ into the centre. For larger MCS, typically with diameters greater than approximately 400–500 μm, the centre is known to become hypoxic^2^ leading to increased rates of glycolysis and consequently increased lactic acid production in their centre. There is also increasing evidence that gene expression in MCS is different to that in 2-D cell culture^3^,^4^,^5^ and is more similar to that found in xenograft tumours compared to 2-D cell culture^6^. In addition to observations of different assay results in 2-D compared to 3-D cell culture^7^,^8^, MCS have been applied to show that certain compounds can be effective at the centres of 3-D MCS but be ineffective at the edges of MCS and in 2-D cell culture^9^. MCS also provide the opportunity to study diffusion rates of a range of substances into and out of the centre of the MCS.

Genetically expressed Förster resonance energy transfer (FRET) biosensors provide a means to measure a wide range of cellular parameters^10^,^11^. When combined with 3-D cell culture, they have been applied to observe changes in redox potential^12^ and the onset of apoptosis^13^ when the MCS are exposed to pharmacological agents, and also to readout AMPK activity^14^. FRET biosensors have also been applied to monitor Rac1, Cdc42 and RhoA activity in a spheroid invasion assay^15^.

The instrumentation available for 3-D imaging in MCS using single-photon fluorescence excitation has been recently reviewed^16^ and multiphoton excitation approaches have also been explored, e.g. refs 4,17, 18, 19, but the speed of these single point scanning approaches are limited by the maximum peak excitation power that can be tolerated by the sample in terms of photobleaching and, in the case of live cells, phototoxicity. Light sheet fluorescence microscopy (LSFM) techniques can overcome this limitation and have also been applied to imaging MCS^12^,^20^,^21^, but conventional LSFM require the MCS to be mounted in a manner that generally precludes high-throughput imaging. This limitation has been addressed using a number of configurations. Galland *et al.*^22^ fabricated custom multiwell plates incorporating a 45° mirror within each well to allow light sheet illumination and imaging with the same objective. However, this approach relies on relatively sophisticated fabrication approaches and is not compatible with conventional multiwell plates. Strnad *et al.*^23^ demonstrated the use of a v-shaped fluorinated ethylene propylene (FEP) channel containing a linear array of 20 zebrafish embryos for time-lapse 3-D imaging of embryo development. While this is a very powerful approach, it does not easily allow different samples within the channel to be exposed to different experimental conditions and the sample preparation process does not easily scale to 100–1000’s samples. Light sheet fluorescence microscopy in conventional multi-well plate arrays has been demonstrated by the use of a fluid filled prism to enable illumination and imaging of the sample at 45° to the plate normal^24^. This is an elegant approach, however the need for an objective with a working distance that is sufficiently long to allow space for the fluid-filled prism limits the NA of the illumination and collection objectives that can be employed. In addition, imaging through the glass coverslip base of the multi-well plate at 45° to the optical axis introduces astigmatism and higher order aberrations that require subsequent correction and which increase in severity as the NA employed increases. High throughput light sheet fluorescence microscopy has also been demonstrated using fluidic approaches where the sample either flows through the light sheet using an FEP tube at 45° to the light sheet^25^, or using a millimetre-scale lab-on-a-chip device fabricated using femtosecond micromachining where the sample flows up through the light sheet towards the detection objective^26^.

This paper concerns the application of LSFM to 3-D imaging of MCS prepared in conventional commercially available 96-well plates on a commercially available inverted microscope frame. In order to deliver the excitation light and collect the fluorescence at 90° using a conventional inverted fluorescence microscope objective and frame, we have adapted the technique of oblique plane microscopy (OPM)^27^ so that it can be used for 3-D imaging via a stage-scanning approach, which we refer to as stage-scanning OPM (ssOPM). OPM uses the same high numerical aperture microscope objective for both illumination and imaging of the specimen without the need for any modifications to the chamber holding the sample. OPM can be considered as a conventional light sheet microscope setup consisting of perpendicular illumination and detection arms where the light sheet is imaged into a remote sample and the resulting fluorescence is returned using a high numerical aperture image relay designed to achieve equal lateral and axial magnifications.

In this paper we demonstrate time-lapse 3-D multi-well imaging of a glucose FRET biosensor in live MCS using ssOPM. We present a range of experiments demonstrating the speed of the system, including imaging 16 fields of view at 10 minute intervals over 4 hours and imaging 42 wells of a 96-well plate in 9 min. Together, these exemplar experiments illustrate the potential of ssOPM to probe a range of experimental questions across a range of different time-scales. Advantages of the full 3-D imaging provided by ssOPM include the ability to calculate the minimum distance to the surface of a spheroid for every voxel, rather than relying on assumptions typically required in the analysis of 2-D images of the spheroid’s sphericity and exact selection of the mid-plane when imaging. We illustrate these advantages by measuring FRET ratio as a function of distance from surface of spheroid for 16 spheroids in a single time-lapse experiment taking 4 hours across 4 different experimental conditions.

## Methods

### OPM

**Figure 1:**
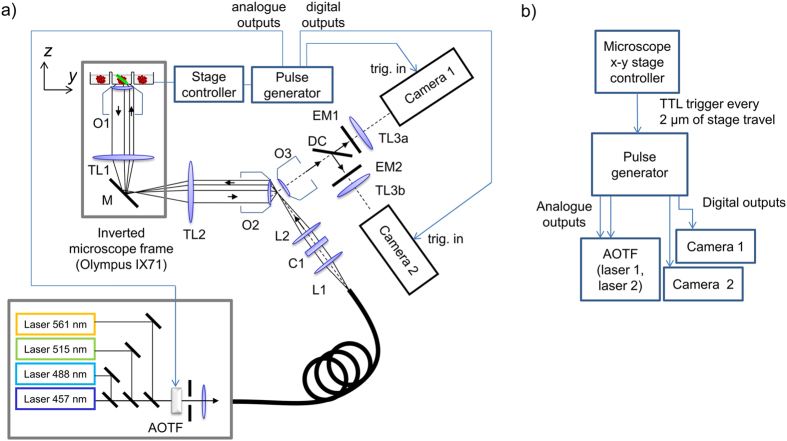
Schematic of ssOPM system. (**a**) system diagram. L – lenses, C – cylindrical lenses, O – microscope objectives, TL – tube lenses, M – mirror, DC – dichroic beamsplitter, EM – emission filter. (**b**) schematic of electrical triggering for image acquisition and laser line control.

The optical setup for OPM, see Fig. 1, has been described previously^27^,^28^,^29^. Briefly, light from four excitation sources at different wavelengths was combined using dichroic mirrors and controlled using an acousto-optic tunable filter (AOTF) before being coupled into a single-mode polarisation maintaining optical fibre. Light exiting the fibre is collimated (L1), focused in the horizontal direction by a cylindrical lens (C1) onto the back focal plane of spherical lens L2, which results in a vertically oriented light sheet angled at 55° to the optical axis of objective lens O2. A pair of microscopes arranged back-to-back (formed by O2, compound tube lens TL2, TL1 and O1) are then used to relay this light sheet to the focal plane of O1, where the light sheet is at 55° to the optical axis of O1. In Fig.1 it is important to note that the optics inside the grey box (inverted microscope frame) are oriented at 90° compared to the rest of the figure, i.e. in the direction perpendicular to the plane of the page. The microscope formed by O1 and TL1 is a commercially available inverted microscope frame (Olympus IX71). O1 is a 60×/1.2NA water immersion objective and so the overall magnification from the focal plane of O1 to the focal plane of O2 is chosen to be equal to the refractive index of water to ensure that the overall lateral and axial magnifications between the focal planes of O1 and O2 are equal, see Botcherby *et al.*^30^ for more detail. The excitation light sheet produced across the focal plane of O1 excites fluorescence in the sample and the resulting fluorescence is imaged back to the focal plane of O2. O3 is positioned so that its optical axis is at 35° to that of O1 and O2 and therefore, together with tube lenses TL3a and TL3b, focuses fluorescence from the excitation light sheet onto cameras 1 and 2. A dichroic beamsplitter DC (Chroma T510lprxtxt-UF3) and emission filters EM1 (Semrock FF01 550/49) and EM2 (Semrock FF01 482/25) separate the emitted fluorescence into two spectral bands for detection of donor and acceptor fluorescence emission respectively. Specifications for all of the optical components, together with a characterisation of the spatial resolution and optical performance of the OPM system can be found in reference^29^.

### ssOPM FRET image acquisition protocol

Stage-scanning OPM was implemented here using a motorized stage (SCAN-IM 120 × 80, Marzhäuser) controlled by a driver unit (Tango 2 fitted with AUX I/O option, Marzhäuser) that can be configured to output a TTL trigger each time the stage has travelled a predefined distance, which was set to 2 μm for the results presented here. This TTL output was connected to a digital acquisition box (National Instruments NI USB-6229) that was configured to output a pattern of signals each time a trigger signal is received from the x-y stage, see Supplementary Figure S1. These signals were used to control the power and duration of the laser excitation and to trigger the start of the exposure of both cameras. The stage was configured to scan in the *y* direction shown in Fig. 1a.

We found that the motorized x-y stage produced mechanical vibrations large enough to cause a reduction in the final image resolution when operated in the speed range 0.4–0.6 μm ms^−1^ and therefore a stage scan speed of 0.1 μm ms^−1^ was used for this work during stage-scan imaging. A faster stage scan speed of 10 μm ms^−1^ was used when translating between fields of view.

As shown in Supplementary Figure S1, after every 2 μm (20 ms) of stage travel the system was configured to provide 2 ms of illumination from the 457 nm laser for excitation of the donor with simultaneous acquisition on both cameras, i.e. providing images of the donor (camera 2) and the sensitised acceptor emission (camera 1). 10 ms later, 2 ms of 515 nm excitation and simultaneous acquisition on camera 1 provided an image of the directly excited acceptor emission. The use of 2 ms camera exposure times means that the sample moves 0.2 μm during the integration, which is less than the measured 0.5 μm in-OPM plane spatial resolution^29^. The details of the laser power used for each experiment are given in Supplementary Table S2.

The two sCMOS cameras (PCO.edge, PCO) were both configured to acquire in Global Reset acquisition mode with 1280×1000 pixels. The exposure time was defined by the 2 ms laser illumination period. To acquire one field of view, the x-y stage was configured to scan 500 μm in the *y* direction (see Fig. 1a), resulting in a stack of 250 images in each of the 3 channels (donor, sensitised emission, directly excited acceptor). Overall, camera 1 was triggered at 100 Hz and camera 2 was triggered at 50 Hz during acquisition. Each 3-D volume took 5 s to acquire and produced 2 GB of raw image data.

Image acquisition was controlled by a HP z800 PC with 96 GB of RAM, 2×512 MB SSD HDD configured in RAID 0 and 8×2TB HDDs configured in RAID 6.

The average background level for each camera when exciting with the 457 nm laser was determined by taking the average pixel value over a blank field of view, which we refer to as *I*_DA background_ for camera 1 (see Fig. 1) and *I*_DD background_ for camera 2.

The inverted microscope frame was fitted with a temperature controlled enclosure set to maintain 37 °C. Microscope objective O1 was fitted with a collar to provide a continuous supply of water immersion liquid and a heating collar set to maintain 37 °C (0280.036, Pecon).

### Image registration

The parameters needed to co-register images acquired on camera 1 and camera 2 (see Fig. 1) were obtained from a single 2-channel fluorescence acquisition of a volume of Sphero Multi-Flurophore 0.13 μm beads (FP-0257-2, Spherotech Inc.) in 10% agarose using the 457 nm excitation laser. These data were then used to determine the *x* shift, *y* shift and rotation needed to co-register data acquired on camera 2 to data acquired on camera 1.

### FRET glucose biosensor

Purified FLII^12^Pglu-700μδ6^31^ ECFP-Citrine glucose FRET biosensor plasmid was received from Addgene (17866, Addgene, UK).

### Cell Culture

HEK293T cells were grown in DMEM medium (31966, Gibco, USA) supplemented with 10% fetal bovine serum (Sigma, UK). Cells were maintained in a humidified incubator set to 37 °C with 5.0% CO_2_.

### Formation of stable cell lines expressing FRET biosensor

To provide a uniform expression level of the biosensor, a clone of stably expressing HEK293T cells was prepared. FLII^12^Pglu-700μδ6 biosensor gene was incorporated into MSCV retroviral vector after restriction digest with HindIII(NEB) and BamH1(NEB) followed by ligation and sequencing. HEK293 cells stably expressing the viral Gagpol gene were transfected with the finished biosensor retroviral plasmid and plasmid for 10A1 envelope protein. Cell supernatant was collected 24 hours later, centrifuged and transferred to HEK293T cells to be targeted with biosensor gene and polybrene (Sigma, UK) added. After 48 hours expression was observed using an epifluorescence microscope. 100 cells were plated sparsely on a 14 cm petri dish and colonies grown for 5 days before selection and expansion.

### Formation of Spheroids

Spheroids were formed using an agarose mould with u-shaped bottomed wells. The Microtissues 12–256 Small Spheroids Kit (Microtissues, USA) was utilised to produce sterile agarose spheroid moulds capable of producing 256 spheroids following the manufacturer’s instructions. Briefly, sterile 4% w/v molten agarose was dispensed into the Microtissues mould, allowed to cool and turned out into a 6 well culture plate. Cells were suspended to a concentration of 1 × 10^6^ ml^−1^ and 190 μl was dispensed into the agarose mould. After 30 minutes, normal growth medium was added to the well to achieve a final volume of 5 ml. 24 hours of incubation were allowed for spheroid formation.

### Imaging medium

Imaging was performed in a home-made medium comprising of 130 mM NaCl, 5 mM KCl, 0.5 mM MgCl, 2 mM CaCl_2_, 10 mM HEPES and 0 mM glucose. D-glucose of the desired concentration was added as indicated in the text. The pH of the medium was adjusted to 7.4 and sterile filtered with a 0.22 μm bottle top filter unit.

### Preparation of 96-well plates

Spheroids were transferred from the agarose mould by inversion into a sterile 50 ml sample tube and placed on ice. After 5 minutes of settling, the volume of medium was reduced to approximately 300 μl by slow aspiration of medium taking care not to disturb spheroids at the bottom of the tube. 100 μl of Matrigel (BD Biosciences; Cat. No. 354234) was added to the spheroids and mixed gently. 25 μl of spheroid/gel mix was pipetted carefully into individual wells of a glass-bottomed 96-well plate (Greiner 96-well SensoPlate 655892) and incubated for 30–45 minutes to form a gel. 125 μl of imaging medium with 25 mM glucose was then added to all wells to give a final volume of 150 μl in each well. For experiments where glucose free conditions were required, medium was subsequently removed and replaced twice with glucose free medium, with 2 minutes incubation between each wash to allow glucose to perfuse out of the gel before replacement. Plates were then placed inside the microscope incubator enclosure and maintained at 37 °C for 1 hour prior to the start of imaging.

### Activator Compounds

When adding glucose to wells initially containing 150 μl glucose free medium, 50 μl of imaging medium with 100 mM glucose was added to a well to reach a final concentration of 25 mM.

The glucose transport inhibitor phloretin (Sigma, UK)^32^,^33^ was used as received and dissolved at a concentration of 400 μM in imaging medium containing 25 mM glucose. 50 μl of this solution was added to a well containing 150 μl of imaging medium with 25 mM glucose to reach a final concentration of 100 μM phloretin without changing the glucose concentration.

β-escin (Sigma, UK), which is a compound causing permeabilisation of the plasma membrane^34^, was dissolved in medium containing glucose and used in experiments to achieve a final concentration of 50 μM β-escin and 25 mM glucose.

### Fluorescent glucose analogue

The fluorescent glucose analogue 2-NBDG (ThermoFisher) was prepared to an initial concentration of 4 mM in imaging medium containing 200 mM glucose. During imaging, 250 μl of this solution was added to 1750 μl of glucose-free imaging medium to reach a final concentration of 500 μM 2-NBDG. Imaging was performed using the 488 nm excitation line and a 500 nm long-pass emission filter (Chroma). Each volume consisted of 250 frames acquired at 50 Hz taking 5 s. Volumes were acquired at 25.5 s intervals.

### Image processing

Raw OPM image data consists of a set of image planes acquired at 55° to the optical axis of O1. It is therefore necessary to transform the data into a conventional coordinate system where *z* is parallel and *x* and *y* are perpendicular to the optical axis of O1. As shown in Fig. 1, the light sheet illumination propagation direction lies in the *y*-*z* plane and the stage motion is in the *y* direction. The reconstruction was performed using a custom-written bi-linear resampling algorithm implemented in MATLAB.

The background-corrected FRET ratio *R*_FRET_ was calculated for each pixel as


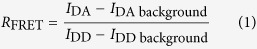


where *I*_DA_ and *I*_DD_ are the signals recorded with excitation at 457 nm with detection in the acceptor and donor channels respectively. *I*_DA background_ and *I*_DD background_ are the background signal levels in these two channels respectively due to the camera offset, camera read noise and background room light, and are determined as described above in the section ‘ssOPM FRET image acquisition protocol’. The colour images presented in this paper were produced using the *parula* colormap bar in MATLAB to represent *R*_FRET_ and with *I*_SE_ represented using brightness.

Due to the use of the CFP-YFP FRET pair in the FLII^12^Pglu-700μδ6 biosensor, there is inevitable bleed-through of donor CFP emission into the acceptor detection channel *I*_DA_ and direct excitation of acceptor YFP by the 457 nm laser. The donor bleed-through and direct excitation of the acceptor are determined by the emission spectra of the fluorophores, the choice of emission filters and dichroic beamsplitter in the detection beam path and the camera spectral sensitivity profile. The effects of donor bleed-through and direct excitation of the acceptor on the calculated FRET ratio are therefore the same for all experiments presented in this paper and therefore all measured FRET ratios can be compared directly.

### Calculation of spheroid minimum distance to surface maps (MDS)

In order to produce a map of the minimum distance to the surface of the spheroid for all points within each spheroid, it was first necessary to determine the location of the spheroid periphery. This was performed by the following procedure:

(1) Threshold all volumes using the same manually determined threshold on *I*_SE_. The threshold was chosen to include the interior of all spheroids within the dataset.

(2) A 3-D binary mask for the interior of the spheroid was then produced by computing a connected component map and selecting the connected component containing the centre of the image volume.

For spheroids where there was incomplete imaging of the top and/or bottom of the spheroid, the following optional steps 3 to 5 were used to estimate the extent of the spheroid by assuming that its shape can be approximated by an ellipsoid of revolution oriented along the *z* axis:

(3) Calculate the total sensitised emission intensity within the spheroid mask for each *z*-plane within the volume.

(4) Fit the resulting curve to an expression for the cross-sectional area *A* perpendicular to the *z* axis of an ellipsoid of revolution as a function of axial position *z*. The ellipsoid of revolution has radius *a* in the *z* axis direction and radius *b* perpendicular to the *z* axis, with an axial centre position *z*_0_, i.e.


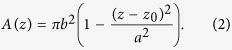


The three fit parameters were *a*, *b* and *z*_0_ and the fit was performed using MATLAB’s *fit* function. The lateral coordinates of the centre of the spheroid (*x*_0_ and *y*_0_) were obtained by calculating the centre of mass of the spheroid mask in the *x* and *y* directions.

(5) The fit parameters obtained in step 4 were used to estimate the cross-sectional area of the spheroid for the top and/or bottom regions that were not captured by the imaging process. A circular mask was then generated for each *z* plane in these regions.

The following step was then applied to all spheroids:

(6) Morphological closure with a radius of 10 μm was then applied to the full mask to fill any holes that may be present within the mask and to smooth the outer boundary. The resulting binary 3-D mask was then eroded with a radius of 6 μm in order to match the visually determined outside perimeter of the spheroid.

For fields of view where a second spheroid was partially imaged at the edge of the field of view and which was included in the mask because of contact between the two spheroids, the mask was manually edited to exclude the second spheroid. When the spheroids are not touching the connected component containing the centre of the image volume is selected by the algorithm (step 2).

For the experiments carried out with the escin cell membrane permeabilisation agent, the spheroids were manually classified as ‘spreading’, i.e. spheroids that have spread out and have a large contact area with the coverslip and that are not well approximated by an ellipsoid of revolution. In this case, only data from the top half of the spheroid were used in the fitting process described in step 4 when estimating the extent of the spheroid above the region imaged. In addition, for spreading spheroids the bottom of the spheroid was assumed to not be in contact with the culture medium when calculating the MDS map.

### Calculation of FRET ratio as a function of MDS

For each time-point for each spheroid a MDS map was calculated. The voxels within each image volume were then binned into MDS intervals of 10 μm and the average FRET ratio was calculated for each interval.

### Data Availability

The raw image data from this study is available under an open source licence from Imperial College London's OMERO server at https://cisbic.bioinformatics.ic.ac.uk/omero/webclient/?show=project-4304.

## Results

### Time-lapse 3-D multi-field of view FRET imaging of MCS in conventional 96-well plates

**Figure 2:**
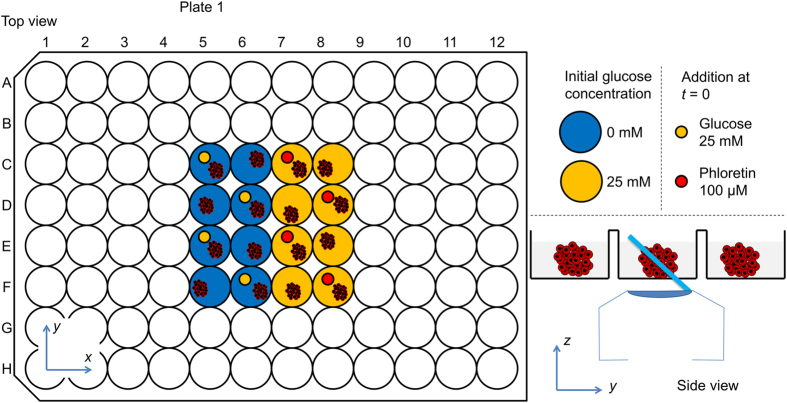
Left, map of plate 1 used for time-lapse 3-D imaging of glucose dynamics in HEK293T MCS. Top right, description of the symbols. Bottom right, side view of plate showing microscope objective and orientation of light sheet within sample.

Plate 1 consisted of a 96-well plate prepared with 16 wells used, see the plate map shown in Fig. 2, with each well containing approximately 10 HEK293T spheroids expressing the FLII^12^Pglu-700μδ6 biosensor. One spheroid per well was manually selected by choosing a spheroid close to the centre of the well and not close to neighbouring spheroids.

**Figure 3:**
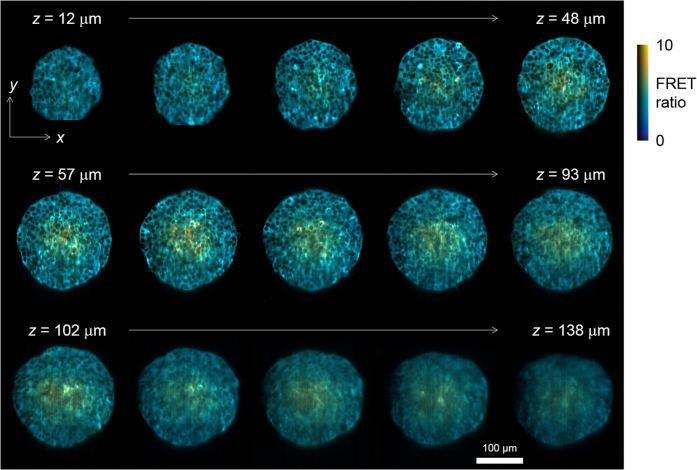
Montage of a sub-set of acquired *z*-planes at 9 μm intervals from a single HEK293T FLII^12^Pglu-700μδ6 spheroid from plate 1 (well C5, see Fig. 2) at time *t* = 174 min. The false-colour scale indicates FRET ratio and brightness indicates the intensity of sensitised emission.

ssOPM was used to acquire 3-D images of all 16 wells in succession, returning to image each well at 10 minute intervals. Image acquisition was started 73 min prior to manual addition of the substances indicated in Fig. 2 and then for a further 174 min after their addition. A total of 24 volumes were acquired for each of the 16 fields of view and the total volume of image data acquired in this experiment was 530 GB. Figure 3 shows the resulting data volume for well C5 imaged at *t* = 174 min. A movie of the full dataset from the same spheroid is shown in Supplementary Video S3. The spatial resolution achieved can be seen to start to degrade for imaging depths >~100 μm which we attribute to scattering of light by the spheroid.

**Figure 4:**
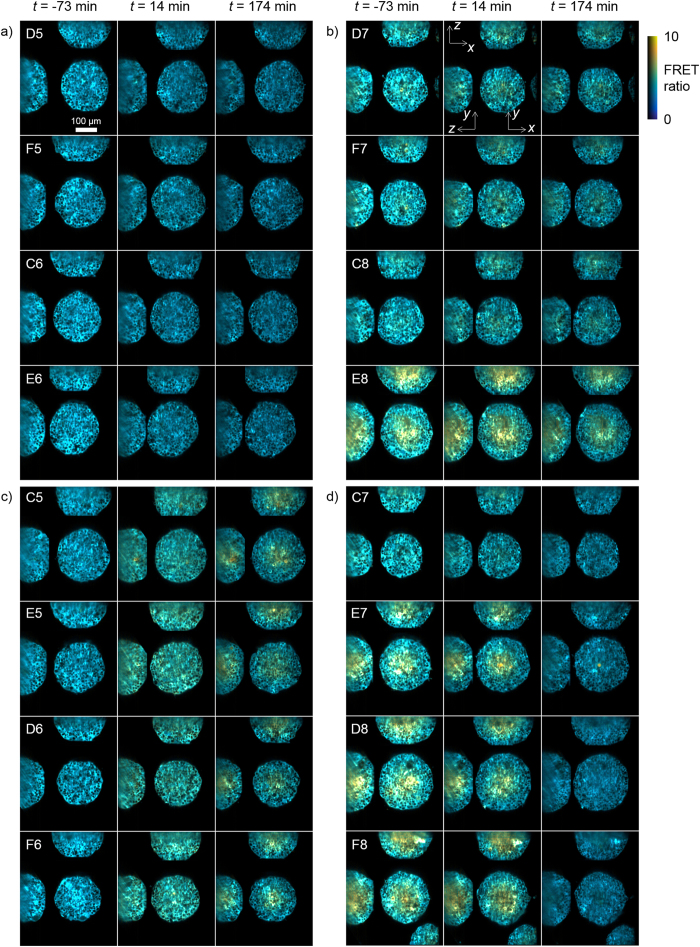
Montage of all HEK293T FLII^12^Pglu-700μδ6 spheroids imaged in plate 1 showing data at t = −73, 14 and 174 min. (a) & (b) panels show data from wells with 0 mM and 25 mM glucose, respectively. Panel (c) shows data from wells initially with 0 mM glucose and addition of 25 mM glucose at *t* = 0. Panel (d) shows data from wells with 25 mM glucose where 100 μM phloretin is added at *t* = 0. An *x*-*y*, *x*-*z* and *y*-*z* slice is shown through the centre of each spheroid. The false-colour scale shows the FRET ratio and brightness indicates intensity of the sensitised emission.

Figure 4 shows a montage of all of the spheroids imaged during this experiment at *t* = −73, 14 and 174 min for the four conditions studied. A movie of the full time-lapse dataset is shown in Supplementary Video S4. The 0 mM glucose control wells (Fig. 4a) exhibited a uniformly low FRET ratio across each spheroid for all time points as expected. For the 25 mM glucose control wells (Fig. 4b), the FRET ratio was higher in the centre of the spheroids than at the edges and the difference was greater for the larger spheroids (e.g. well E8). For the wells initially without glucose with 25 mM glucose added at *t* = 0, see Fig. 4c, the FRET ratio is uniformly low at *t* = −73 min. At *t* = 14 min there is an approximately uniform increase in FRET ratio across each spheroid. At *t* = 174 min there is a higher glucose concentration in the centre of each spheroid compared to the edges. For the wells initially exposed to 25 mM glucose and with 100 μM phloretin added at *t* = 0, the spheroids initially show similar spatial variation in FRET ratio to the 25 mM glucose control wells. Following addition of phloretin – a glucose transport inhibitor^32^,^33^ – the FRET ratio gradually decreases to a uniformly low value consistent with that seen in the 0 mM glucose control wells.

### Analysis of HEK293T FLII^12^Pglu-700μδ6 3-D data

**Figure 5:**
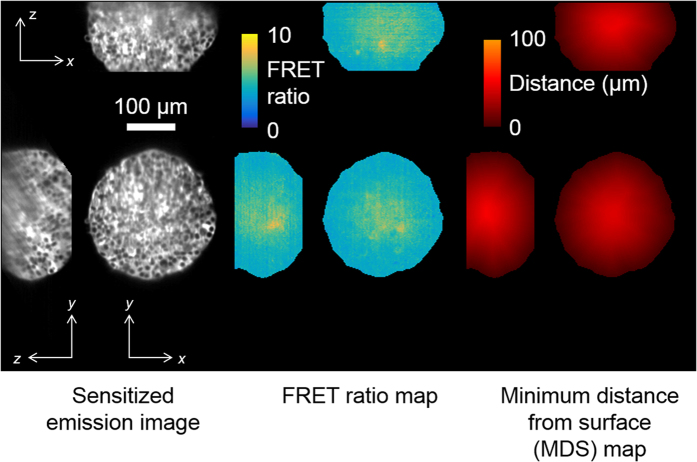
Left column, sensitised acceptor emission intensity for *x*-*y*, *x*-*z* and *y*-*z* slices through the centre of the HEK293T FLII^12^Pglu-700μδ6 spheroid imaged in well C5 of plate 1 at *t* = 174 min. Middle column, corresponding FRET ratio map without intensity merging. Right column, calculated map of the minimum distance to the surface of the spheroid.

As the FRET ratios observed vary with distance from the surface of the spheroid, 3-D minimum distance to surface (MDS) maps were calculated for each spheroid at each time-point, see Materials and Methods. Example 3-way cuts of the resulting MDS maps for wells C5 of plate 1 are shown in Fig. 5.

**Figure 6:**
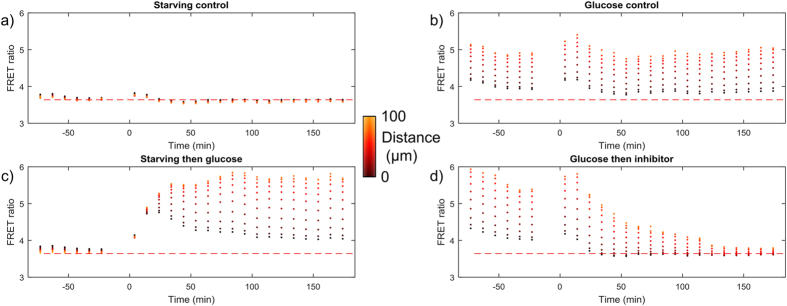
HEK293T FLII^12^Pglu-700μδ6 FRET ratio as a function of MDS averaged over all four spheroids for each condition. (**a**) 0 mM extracellular glucose control, (**b**) 25 mM glucose control, (**c**) 0 mM glucose then 25 mM glucose and (**d**) 25 mM glucose then addition of 100 μM phloretin. The horizontal dashed red line shows the mean FRET ratio over all time-points for panel (a).

The voxels within each spheroid were then binned into 10 μm MDS intervals allowing the mean FRET ratio to be calculated for each interval, see Fig. 6. The control data from spheroids exposed to zero glucose for all time points (wells C6, D5, E6 and F5) show that the FRET ratio does not vary with MDS or time and is uniformly low. The data from spheroids exposed to 25 mM glucose for all time points (wells C8, D7, E8 and F7) show a higher FRET ratio in the centre compared to the edges, see Discussion section. There is a small ripple in the FRET ratio values around *t* = 0 for both the zero and 25 mM glucose wells, which we attribute to the change in temperature within the microscope enclosure caused by opening the door in order to manually add the substances indicated in Fig. 2. The quantum efficiency of eCFP, which is the donor in the FLII^12^Pglu-700μδ6 biosensor, has previously been shown to be sensitive to temperature^35^.

For spheroids initially exposed to 0 mM glucose and with 25 mM glucose added at *t* = 0 (wells C5, D6, E5 and F6), the FRET ratio initially increases uniformly for all MDS values. From *t* = ~20 min onwards, the FRET ratio at the surface of the spheroids overshoots the final steady-state value while the FRET ratio for larger MDS values gradually saturates to a higher value. This can be seen in all 4 spheroids for this condition individually, see Supplementary Figure S5. For spheroids initially exposed to 25 mM glucose with 100 μM phloretin added at *t* = 0 (wells C7, D8, E7 and F8), for *t* < 0 the data shows that the FRET ratio in the centre of the spheroid is higher than the edges, which is consistent with the glucose control data (wells C8, D7, E8 and F7). For times *t* > 0 the FRET ratio falls with time down to the level seen in the in the zero glucose control data (wells C6, D5, E6 and F5).

The results presented for spheroids initially exposed to zero glucose and then with 25 mM glucose added at *t* = 0 and for spheroids initially exposed to 25 mM glucose with 100 μM phloretin added at *t* = 0 were repeated independently on a separate plate (plate 2) on a different day again with four spheroids per condition. Similar trends were observed, see Supplementary Figure S6. The inter- and intra-plate variation in FRET ratio is investigated in more detail below.

**Figure 7:**
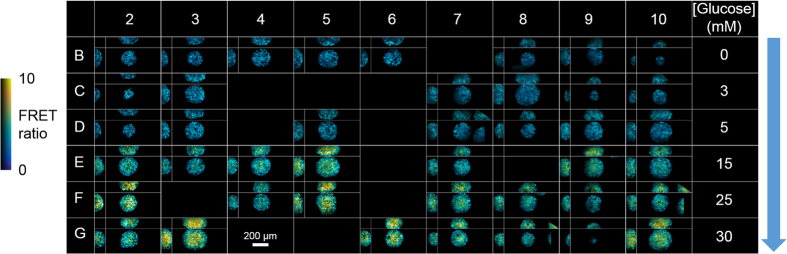
Demonstration of ssOPM plate-reading capability on 42 wells for culture media with varying glucose concentration (plate 3). Glucose concentration in the culture medium increases down the plate as indicated on the right hand side. For each well, an *x*-*z* (top), *y*-*z* (left) and *x*-*y* (bottom right) slice is shown through the spheroid.

**Figure 8:**
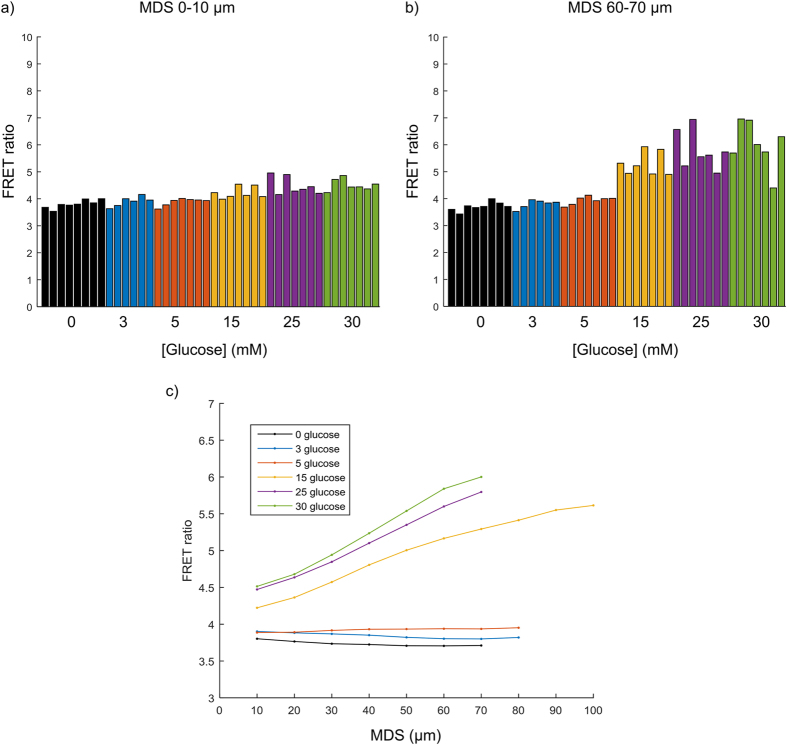
Quantification of FRET ratio for glucose titration (plate 3). (**a**) FRET ratio for the surface of each spheroid (MDS range 0-10 μm). (**b**) FRET ratio for an inner region of each spheroid (MDS range 60–70 μm). (**c**) Plot of mean FRET ratio across repeat wells as a function of MDS for all glucose concentrations.

To demonstrate the potential for the ssOPM to image a larger number of fields of view automatically, we prepared a 96-well plate (plate 3) where spheroids were seeded into 54 wells. Six different glucose concentrations in the culture medium ranging from 0 to 30 mM were applied across the plate with 9 replicate wells per condition. Spheroids could only be located close to the coverslip in 42 of these wells. The total acquisition time for 42 fields of view including translation of the stage between fields was 9 minutes and the FRET ratio images are shown in Fig. 7. An MDS analysis of these data is presented in Fig. 8, which shows that there is a gradual increase in FRET ratio for the surface of the spheroids with increasing concentration of glucose in the culture medium. For inner regions of the spheroids this increase is more pronounced and has greater heterogeneity.

We compared the FRET ratios obtained at the first time-point from all three plates described above plus the results from a further experiment (plate 4), which were all prepared and imaged on different days. The data are shown as a bar chart in Supplementary Figure S7 and the standard deviation across all measurements and the intra- and inter-plate standard deviations are shown in Table 1. The standard deviation in FRET ratio was lowest for the outside of the spheroids (MDS 0–10 μm) with 0 mM glucose in the culture medium for all three measures of standard deviation. The standard deviations were larger in the presence of 25 mM glucose in the culture medium. For the interior of the spheroids (MDS 60–70 μm) the standard deviations were larger than for the outside but followed similar trends.

In order to establish the dynamic range of the FLII^12^Pglu-700μδ6 biosensor on the ssOPM system, we performed time-lapse imaging of spheroids in a culture medium containing 25 mM glucose where 50 μM β-escin – a compound causing permeabilisation of the plasma membrane^34^ – was added at time *t* = 0, see Supplementary Figure S8. In spheroid 1 the FRET ratio for the outside of the spheroid (MDS 0–10 μm) saturated at 10.3 and for spheroid 3 the FRET ratio saturated at 12.3. For spheroid 2 a plateau was not obtained, but the maximum value recorded was 13. We attribute the variation in response to differences in cell permeabilisation by escin in the different repeats causing differences in the glucose influx rate relative to cellular glucose metabolism.

Finally, to check that the dynamics of the glucose response seen in Fig. 6 are not determined by the rate at which glucose diffuses into the intercellular (interstitial) space between the cells forming the spheroid, we applied ssOPM imaging to image diffusion of the fluorescent glucose analogue 2-NBDG as it is added to the culture medium of a spheroid that has been starved of glucose. Supplementary Figure S9 shows that the intercellular glucose concentration (measured in a region of interest inside the spheroids) plateaus in ~5 min for 3 separate repeats of the experiment.

## Discussion

The results obtained from HEK293T MCS expressing the FLII^12^Pglu-700μδ6 biosensor revealed that spheroids cultured in the presence of glucose have a higher FRET ratio in the centre of the spheroid compared to the edges, see Fig. 4b. Although cells are generally thought to be able to maintain a tight regulation of intracellular pH even when metabolically generating an extracellular acidification, we cannot exclude an intracellular acidification at the core of the spheroids^36^, which may be partially responsible for the change in the FRET ratio observed. This is because both the donor eCFP and acceptor Citrine in the FLII^12^Pglu-700μδ6 biosensor used here are known to be sensitive to pH^37^,^38^. Therefore, the increase in FRET ratio in the centres of the spheroids in the presence of glucose in the culture medium may be due to a pH gradient within the spheroid rather than an intracellular glucose gradient. In order to investigate this observation further, the next step would be to perform repeat measurements using a near-identical biosensor with no or very low affinity for glucose as a negative control^39^.

Cells at the edges of the spheroids in our experiments are expected to experience the well-defined extracellular pH of the culture medium (pH 7.4) and therefore for these cells changes in observed FRET ratio can be ascribed to changes in intracellular glucose with greater confidence. Previous work has shown that HEK293T cells show a low response to external glucose and have a low endogenous glucose transport activity^39^. This is consistent with the results that we obtain for the outside of the spheroids, see Fig. 8a, i.e. even in the presence of high glucose in the culture medium the increase in FRET ratio is small compared to saturation of the sensor (Supplementary Figure S8).

For spheroids initially exposed to 0 mM glucose that then have glucose added, see Figs 4c and 6c, the initial increase in FRET ratio is approximately uniform across the spheroids until at least *t* = ~20 min. After this time, the FRET ratio for the outside of the spheroid decreases slightly and plateaus whereas the FRET ratio for the interior of the spheroid continues to increase and plateaus at a higher value. This difference in biosensor response could be due to differences in gene expression, e.g. of plasma membrane glucose transporter proteins, causing different intracellular glucose uptake dynamics for cells in the centre of the spheroid relative to those on the outside. We note that prior to the experiment, the spheroids were cultured in 25 mM glucose and so would have time to establish different protein expression levels due to different local extracellular environments with distance into the spheroid. Another possibility is that the addition of glucose causes a pH gradient to be set up in the spheroid that affects the readout of the biosensor used dynamically over time. Further experiments are required to investigate this fully. Our results obtained using the fluorescent glucose analogue 2-NBDG suggest that diffusion of glucose into the centre of the spheroid is relatively fast (plateaus in ~5 min, Supplementary Figure S9), and therefore the difference in FRET ratio dynamics seen for the inside and outside of the spheroid are not likely to be due to the difference in local extracellular glucose concentration.

We compared the inter- and intra-plate variability in FRET ratio for the MDS ranges 0–10 and 60–70 μm (see Supplementary Figure S7). Our results show that the FRET ratio for spheroids cultured in the absence of glucose is the most consistent both within and between plates, see Table 1. For spheroids cultured in the presence of 25 mM glucose, in the MDS range 0–10 μm the variation in FRET ratio was higher than for 0 mM glucose, and the variation increased further for the MDS range 60–70 μm. These trends can also be seen in Fig. 8. Cells exposed to 0 mM are likely to have a uniformly low intracellular glucose and therefore the least biological heterogeneity. Cells cultured with glucose or that are within the spheroid environment are likely to have the greater heterogeneity in local environment.

In the control wells of our time-lapse experiment (plate 1, Fig. 4 a&b), we observed a variation in FRET ratio around *t* = 0 that we attribute to the change in temperature caused by opening the door of the microscope enclosure in order to add aliquots to the wells. In the future, any temperature change can be avoided by using a system for remote addition of compounds to the plate and/or the use of an improved biosensor with reduced sensitivity to temperature.

Our experimental protocol acquires data in three channels (donor, sensitised emission and directly excited acceptor). In the future, so-called 3-cube FRET calculations^40^ can therefore be applied to ssOPM data enabling more quantitative measurements of the status of the biosensor. This can be achieved in future by determining the necessary correction factors using samples expressing the appropriate control constructs^41^.

Our ssOPM system is currently not able to image wells on the outermost edge of a 96-well plate due to the collar supplying water immersion liquid to the tip of the microscope objective obstructing the movement of the motorised stage. In the future, this problem can be avoided by modifying the mechanical design of the collar or the stage insert.

## Conclusions

We have demonstrated the potential of ssOPM to probe spatio-temporal dynamics in 3-D in MCS expressing a FRET biosensor in commercially available glass-bottomed 96-well plates. We used HEK293T cells expressing the FLII^12^Pglu-700μδ6 intercellular glucose biosensor read out using spectral ratiometric measurements. Sub-cellular resolution imaging was obtained for imaging depths of ~100 μm into a spheroid. We imaged 16 wells at 10 minute intervals for 4 hours generating 530 GB of raw image data (plate 1). An independent repeat of this experiment on a plate prepared and imaged on different days illustrates the repeatability of the method (plate 2). We illustrated the potential of ssOPM to perform a glucose dose-response measurement in 3-D in a 96-well plate using 42 wells that required 9 min for image acquisition (plate 3).

Our 3-D imaging approach revealed an increase in FRET ratio in the centre of spheroids cultured in the presence of glucose. This may be due to gradients of glucose or pH within the spheroid and further experiments and/or the use of an improved biosensor is required to investigate this further. In addition, ssOPM was used to record the spatio-temporal FRET ratio response as glucose-starved HEK293T spheroids were exposed to glucose. Using the 3-D image data acquired, we calculated the FRET ratio as a function of the minimum distance of an image point to the surface of the spheroid as a function of time. In the future, ssOPM may find application to studying a wide variety of FRET biosensors in 3-D cell cultures across a wide-range of conditions in commercially available 96-well plates.

## Additional Information

**How to cite this article**: Maioli, V. *et al.* Time-lapse 3D measurements of a glucose biosensor in multicellular spheroids by light sheet fluorescence microscopy in commercial 96-well plates. *Sci. Rep.* **6**, 37777; doi: 10.1038/srep37777 (2016).

**Publisher's note:** Springer Nature remains neutral with regard to jurisdictional claims in published maps and institutional affiliations.

## Supplementary Material

Supplementary Information

Supplementary Video S3

Supplementary Video S4

## Figures and Tables

**Table 1:** Standard deviation in measured FRET ratio for different MDS values and glucose concentration in culture medium for: all spheroids, intra-plate and inter-plate.

MDS (μm)	Glucose concentration in culture medium (mM)	Standard deviation in FRET ratio		
		Across all spheroids	Intra-plate	Inter-plate
0–10	0	0.12	0.07	0.09
	25	0.25	0.21	0.16
60–70	0	0.13	0.08	0.1
	25	0.75	0.55	0.61
