# Supplementary material for: Time-lapse 3-D measurements of a glucose biosensor in multicellular spheroids by light sheet fluorescence microscopy in commercial 96-well plates
Source: Sci Rep. 2016 Nov 25;6:37777. doi: 10.1038/srep37777 (PMC5122899; doi:10.1038/srep37777)

Time-lapse 3-D measurements of a glucose biosensor in multicellular spheroids by light sheet fluorescence microscopy in commercial 96-well plates

Supplementary information

Vincent Maioli[1], George Chennell[1,2,3], Hugh Sparks[1], Tobia Lana[2,3], Sunil Kumar[1], David Carling[2,3], Alessandro Sardini[2,3], Chris Dunsby[1,4]

[1] Photonics Group, Department of Physics, Imperial College London

[2] MRC Clinical Sciences Centre (CSC)

[3] Institute of Clinical Sciences (ICS), Faculty of Medicine, Imperial College London

[4] Centre for Pathology, Faculty of Medicine, Imperial College London

Supplementary Figure S1. Timing diagram for spectral ratiometric FRET image acquisition. Every 20 ms the translation stage sends a trigger which initiates image acquisition on both cameras with 457 nm excitation, with a third image recorded on camera 1 with 515 nm excitation 10 ms later.

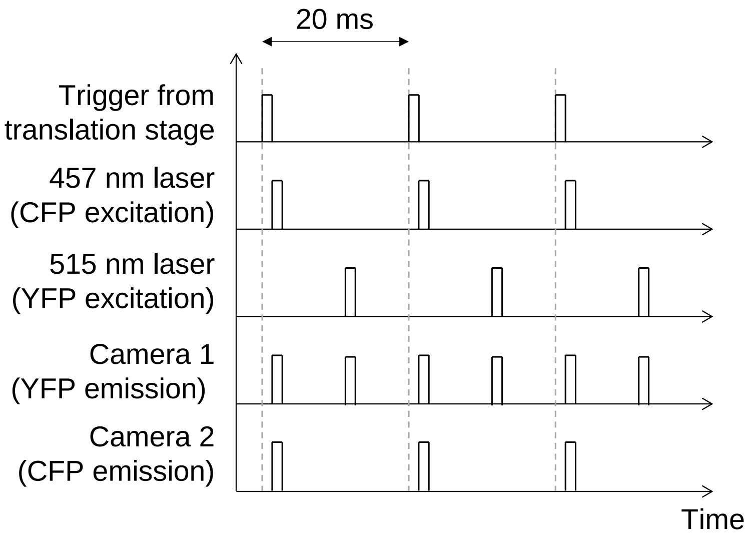

Supplementary Table S2. Summary of laser power and light doses at the beam waist for the different experiments. The parameters used were exposure time 2 ms, light sheet thickness 3.8 µm[27], system FOV 400 µm.

	457 nm excitation wavelength		515 nm excitation wavelength	
Experiment	Power at BFP (µW)	Radiant exposure per image ($J.m^{-2}$)	Power at BFP (µW)	Radiant exposure per image ($J.m^{-2}$)
Plate 1	560	740	140	180
Plate 2	560	740	140	180
Plate 3	370	490	130	170
Permeabilisation	560	740	140	180

Supplementary Video S3. Time-lapse video of a montage of a sub-set of acquired z-planes at 9 µm intervals of the spheroid from well C5 of plate 1, same as shown in Figure 3. The spheroid was initially starving and 25 mM glucose was added to the medium at t = 0.

Supplementary Video S4. Time-lapse video of a montage of three-way cuts of all spheroids from Plate 1.

Supplementary Figure S5. From Plate 1, HEK293T FLII^{12}Pglu-700µδ6 FRET ratio as a function of MDS for all four spheroids for the 0 mM glucose then 25 mM glucose condition. The horizontal dashed line shows the mean FRET ratio over all time-points from Figure 6 (a).

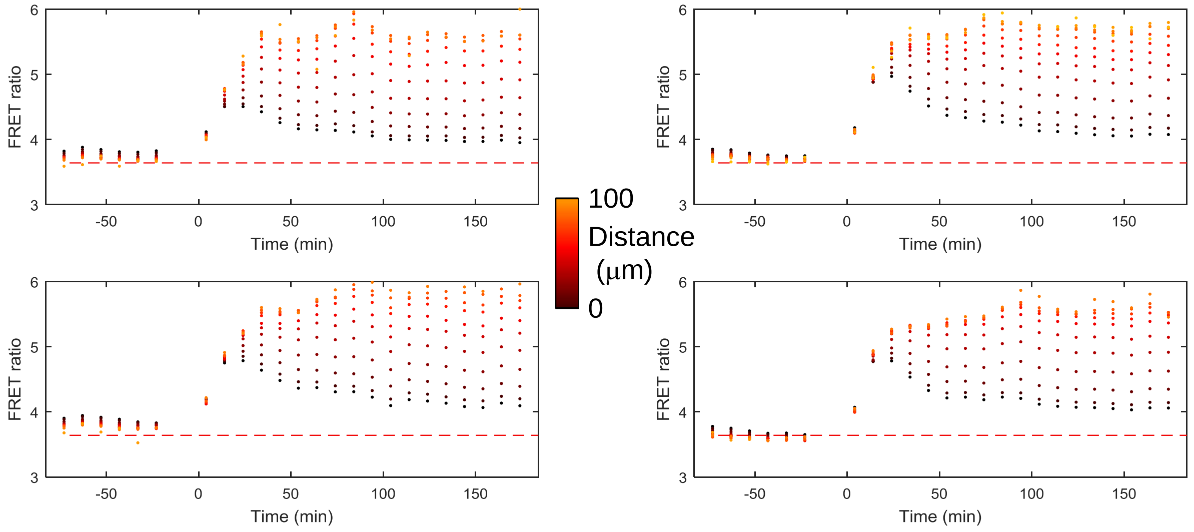

Supplementary Figure S6. From Plate 2, HEK293T FLII^{12}Pglu-700µδ6 FRET ratio as a function of MDS averaged over all four spheroids for each condition: a) 0 mM glucose then 25 mM glucose and b) 25 mM glucose then addition of 100 µM phloretin. The horizontal dashed line shows the mean FRET ratio over the first six time-points for panel (a).

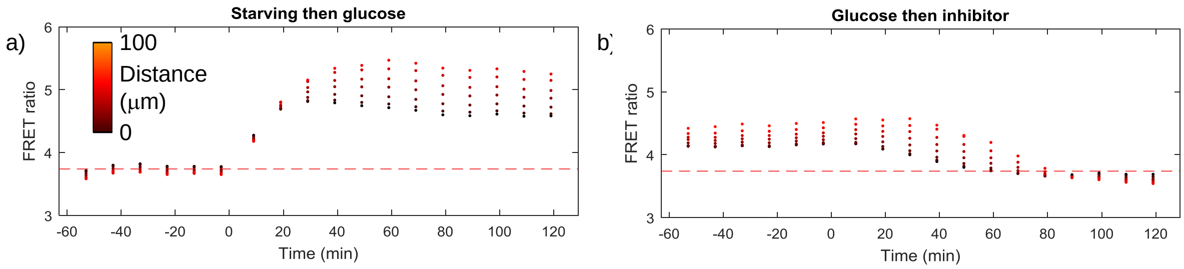

Supplementary Figure S7. HEK293T FLII^{12}Pglu-700µδ6 FRET ratio for two conditions and two MDS for the four plates imaged. (a) No glucose, MDS 0-10 µm. (b) 25 mM glucose, MDS 0-10 µm. (c) No glucose, MDS 60-70 µm. (c) 25 mM glucose, MDS 60-70 µm.

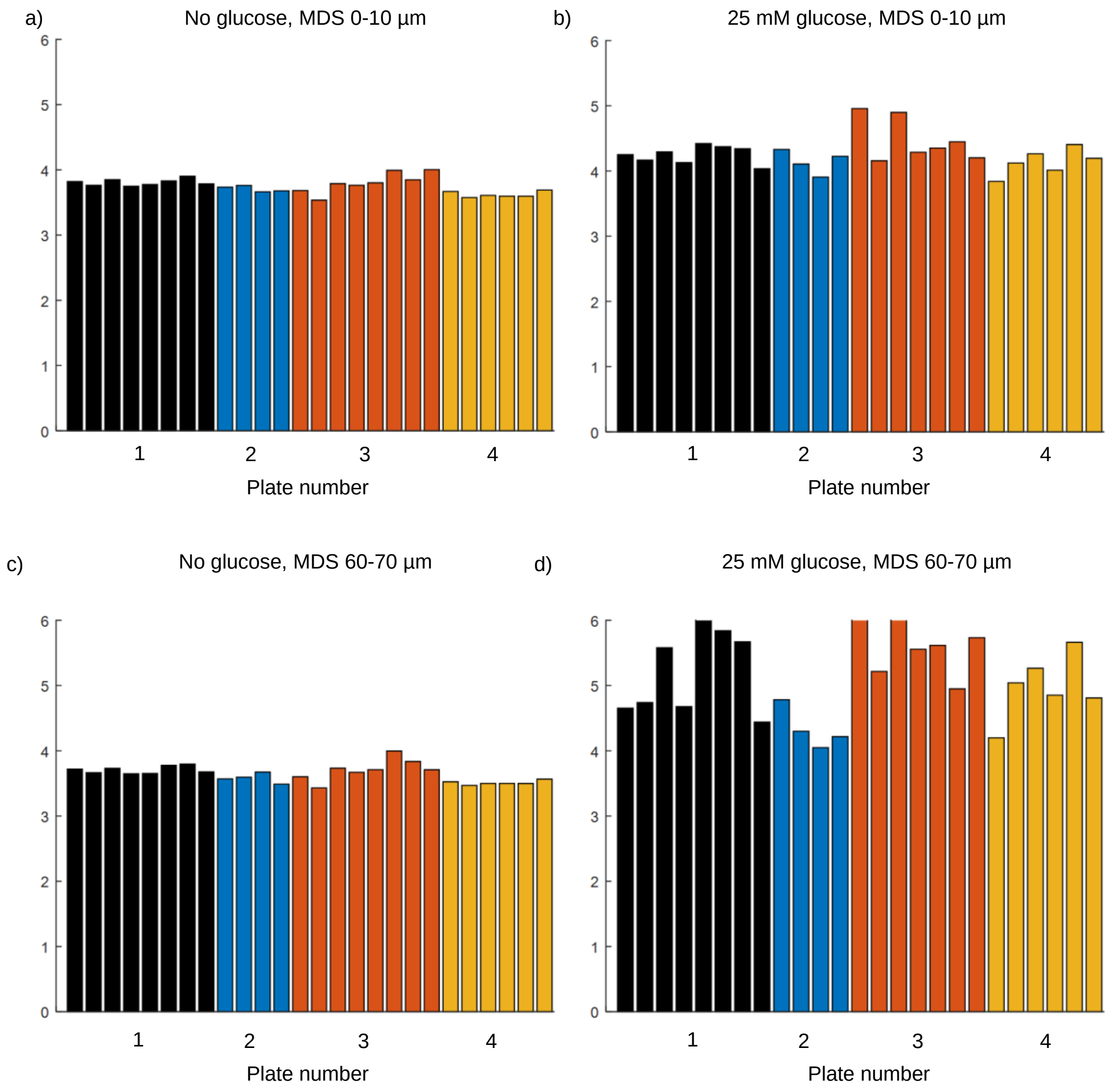

Supplementary Figure S8. Effect of escin membrane permeabilisation on HEK293T FLII^{12}Pglu-700µδ6 spheroids cultured with 25 mM glucose. (a) Three-way cuts of the three spheroids at *t* = -40 min and *t* = 5 min. (b) Quantification of the FRET ratio as a function of time and MDS.

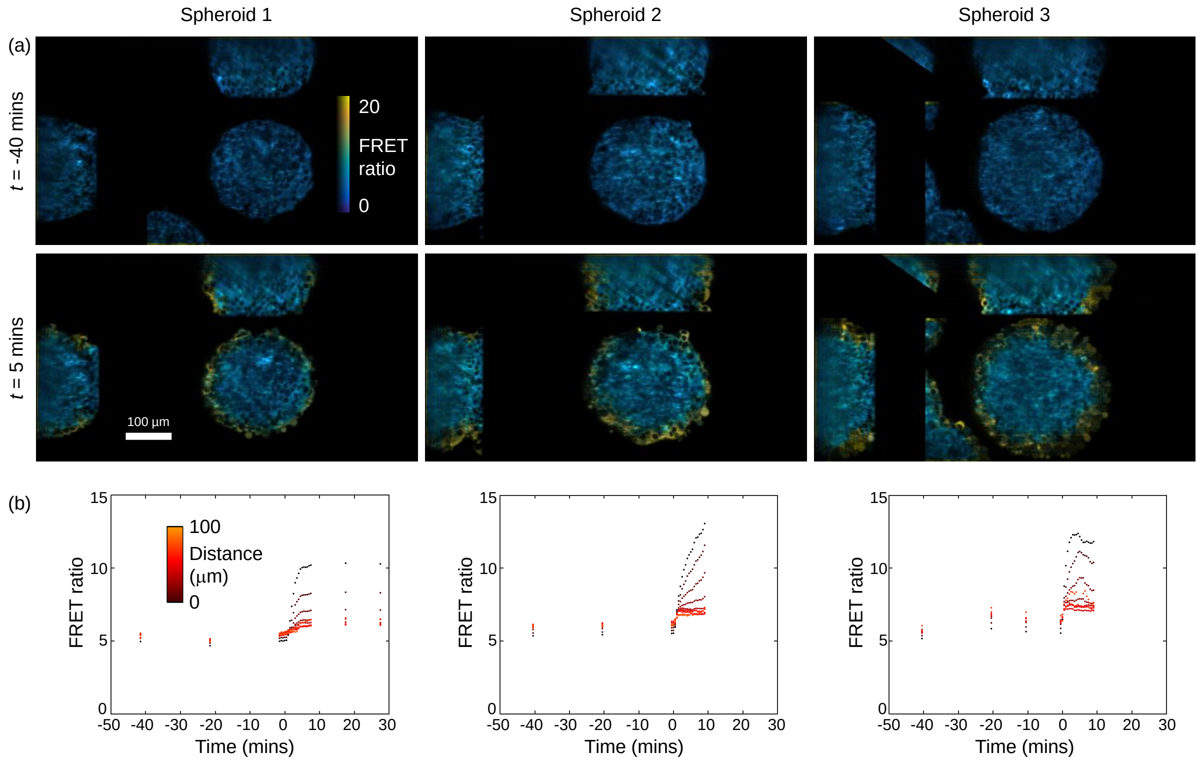

Supplementary Figure S9. Diffusion of 2-NBDG into the interstitial space between cells within the spheroids. (a) three-way cuts of data obtained from Spheroid 1 (b) quantification of 2-NBDG fluorescence in the centre of the spheroids (blue) and outside the spheroids (green).

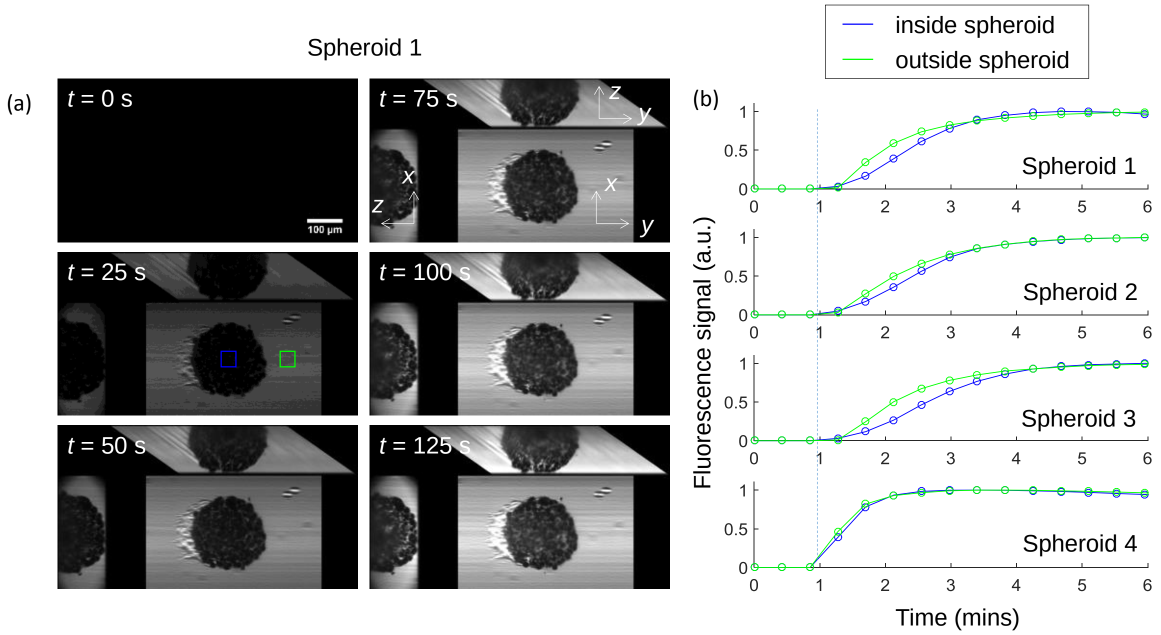

Supplement: Supplementary Information [file srep37777-s1.pdf]
